# Ultrasounds induce blood–brain barrier opening across a sonolucent polyolefin plate in an in vitro isolated brain preparation

**DOI:** 10.1038/s41598-022-06791-7

**Published:** 2022-02-21

**Authors:** Laura Librizzi, Laura Uva, Luca Raspagliesi, Matteo Gionso, Maria Cristina Regondi, Giovanni Durando, Francesco DiMeco, Marco de Curtis, Francesco Prada

**Affiliations:** 1 Epilepsy Unit, Fondazione IRCCS Istituto Neurologico Carlo Besta, Milan, Italy; 2 Department of Neurosurgery, Humanitas University, Milan, Italy; 3 Department of Biomedical Sciences and Humanitas Clinical and Research Center, IRCCS, Milan, Italy; 4 Acoustic Neuroimaging and Therapy Laboratory, Fondazione IRCCS Istituto Neurologico Carlo Besta, Milan, Italy; 5 Department of Neurological Surgery, Fondazione IRCCS Istituto Neurologico Carlo Besta, Milan, Italy; 6 Ultrasound Laboratory, Istituto Nazionale di Ricerca Metrologica I.N.Ri.M., Turin, Italy; 7 Department of Neurological Surgery, Johns Hopkins Medical School, Baltimore, MD USA; 8 Department of Health Sciences, University of Milan, Milan, Italy; 9 Department of Neurological Surgery, University of Virginia Health System, Charlottesville, VA USA; 10 Focused Ultrasound Foundation, Charlottesville, VA USA

**Keywords:** CNS cancer, Blood-brain barrier, Biomedical engineering, Diseases of the nervous system

## Abstract

The blood–brain barrier (BBB) represents a major obstacle to the delivery of drugs to the central nervous system. The combined use of low-intensity pulsed ultrasound waves and intravascular microbubbles (MB) represents a promising solution to this issue, allowing reversible disruption of the barrier. In this study, we evaluate the feasibility of BBB opening through a biocompatible, polyolefin-based plate in an in vitro whole brain model. Twelve in vitro guinea pig brains were employed; brains were insonated using a planar transducer with or without interposing the polyolefin plate during arterial infusion of MB. Circulating MBs were visualized with an ultrasonographic device with a linear probe. BBB permeabilization was assessed by quantifying at confocal microscopy the extravasation of FITC-albumin perfused after each treatment. US-treated brains displayed BBB permeabilization exclusively in the volume under the US beam; no significant differences were observed between brains insonated with or without the polyolefin plate. Control brains not perfused with MB did not show signs of FITC-albumin extravasation. Our preclinical study suggests that polyolefin cranial plate could be implanted as a skull replacement to maintain craniotomic windows and perform post-surgical repeated BBB opening with ultrasound guidance to deliver therapeutic agents to the central nervous system.

## Introduction

The blood–brain barrier (BBB) represents the interface between cerebral vessels and the central nervous system that maintains cerebral homeostasis and protects the brain from circulating agents^1^. BBB is composed of a layered structure of endothelial cells, basal membrane, mural cells, pericytes and astrocytic glial cells^2^; its mechanical selectivity depends greatly on the lack of capillary fenestrae and on the tight junctions between adjacent endothelial cells, which provide a high-resistance and size-selective filter preventing most of the systemically applied pharmacological agents from having therapeutic effects in the brain^1,3^.

It is essential to overcome this relevant barrier to deliver therapies for neurological diseases. In this regard, BBB permeabilization obtained through low-frequency, low-intensity ultrasound (US) waves combined with intravenously injected microbubbles (MBs) has gained particular interest, as it is delivered in a localized, reversible, and non-invasive fashion compared to other procedures^3,4^. MBs are high-molecular weight gasses encapsulated in either lipid, protein or polymeric shells, approximately the size of an average red blood cell^5,6^; they were originally designed for contrast-enhanced ultrasound (CEUS) imaging, as they are purely intravascular, highly echogenic and allow excellent macro and micro-vasculature visualization^7,8^. MBs represent a key component of US-mediated BBB opening, as they act as intravascular “cavitation nuclei”, concentrating the energy and effects of the intersecting US waves to the vessels wall, reducing the power of insonation and thus sparing the surrounding parenchyma^9^.

US-induced BBB opening relies on the phenomenon of acoustic cavitation: the micro-oscillation of gasses within vessels induced by an intersecting US beam loosens the intercellular junctions of the vessel walls to transiently increase BBB permeability^4,10^. The resulting temporary BBB impairment allows greater paracellular passage of substances and drugs into the brain parenchyma in a restricted and controlled time window^4,11^.

Different setups have been developed and employed, such as implantable US transducers, MR-guided US suites and navigated US devices^12–15^. Although mostly experimental, such endeavors have already produced remarkable results in both preclinical settings and preliminary clinical experiences^12–15^. However, their broad clinical application is hindered by many factors: on one hand, available devices rely on the modality of focused ultrasound (FUS), in which US beams are concentrated in a single spot of restricted size to achieve the highest accuracy and precision of insonation; such concept unavoidably has an impact on both the volume size and the total time of treatment, making larger targets such as tumours hardly treatable, especially for wide cohorts of patients. On the other hand, currently available systems track the treatment based on either MRI/TC images^12–15^—pre-acquired or obtained during insonation itself—or the fixed radius of an implanted therapeutical US (TUS) device with no direct imaging feedback^16,17^. Another limitation of current systems is that MBs distributions during both planning of the treatment and insonation itself cannot be visualized because of the high impedance of the skull bone that prevents the transcranial MBs imaging. However, a dependence of BBB permeabilization outcome on MBs concentration and distribution within tissue could be hypothesized^9,18,19^. CEUS imaging obtained through a craniotomic access, allows to investigate such phenomena^3,4^. Ideally, the implant of a sonolucent cranial protheses in those patients requiring a craniotomy could facilitate the evaluation of MB dynamic concentration during repeated therapeutic BBB disruption treatments, further tailoring the procedure.

As a matter of fact skull replacements are routinely used in neurosurgery for multiple purposes, encompassing the repair of congenital or acquired malformations, the reconstruction of large cranial defects, the resorption of skull bones as late complications of craniotomy procedures or oncological radicality; indeed, conditions associated with cranioplasty procedures include trauma, infection, stroke and brain tumors^20^. Of note, recent papers report advantages of using synthetic prostheses rather than autologous skull bone, such as lower rates of reoperation and local complications; however, most of the literature focuses on cranioplasty following decompressive craniectomy—regardless the cause—in which conservation processes are implied that do not subsist in elective procedures^21,22^.

The proposed approach entails a biocompatible polyolefin-based skull prosthesis to be implanted in selected patients at the end of the surgery instead of the native bone and exploited subsequently as a sonolucent window to perform both ultrasonographical post-surgical follow-up and ultrasonography-guided BBB opening treatments^23^. Our approach envisages the use of a 4 mm-thick polyolefin-based cranial prosthesis (In.Tra., Milan, Italy, PCT/EP2014/068837, human biocompatibility ISO-10993) that was developed and patented to allow transcranial US imaging and therapy; this material has been recently tested in vitro and in vivo to demonstrate its competence to minimize attenuation and deviation of the US waves and to validate its use for trans-prosthesis imaging^23^.

The aim of the present study was to demonstrate the feasibility of MB enhanced TUS-mediated BBB opening through a polyolefin plate. To this end, the study took advantage of the isolated guinea pig brain maintained in vitro by arterial perfusion, a unique experimental preparation in which the BBB as well as vascular and neuronal compartments are morphologically and functionally preserved for several hours^24–26^. BBB opening is achieved by means of an unfocused US transducer applied over one brain hemisphere after perfusion with MBs had been verified through CEUS imaging. The increase in BBB permeability obtained in the presence of the polyolefin plate was compared in brains insonated without the interposition of the device. Our preclinical findings demonstrate that polyolefin plates could be used as cranial prostheses to perform repetitive focal BBB openings in clinical settings.

## Results

### Acoustic characterization of the ultrasound-transparent polyolefin plate

**Figure 1:**
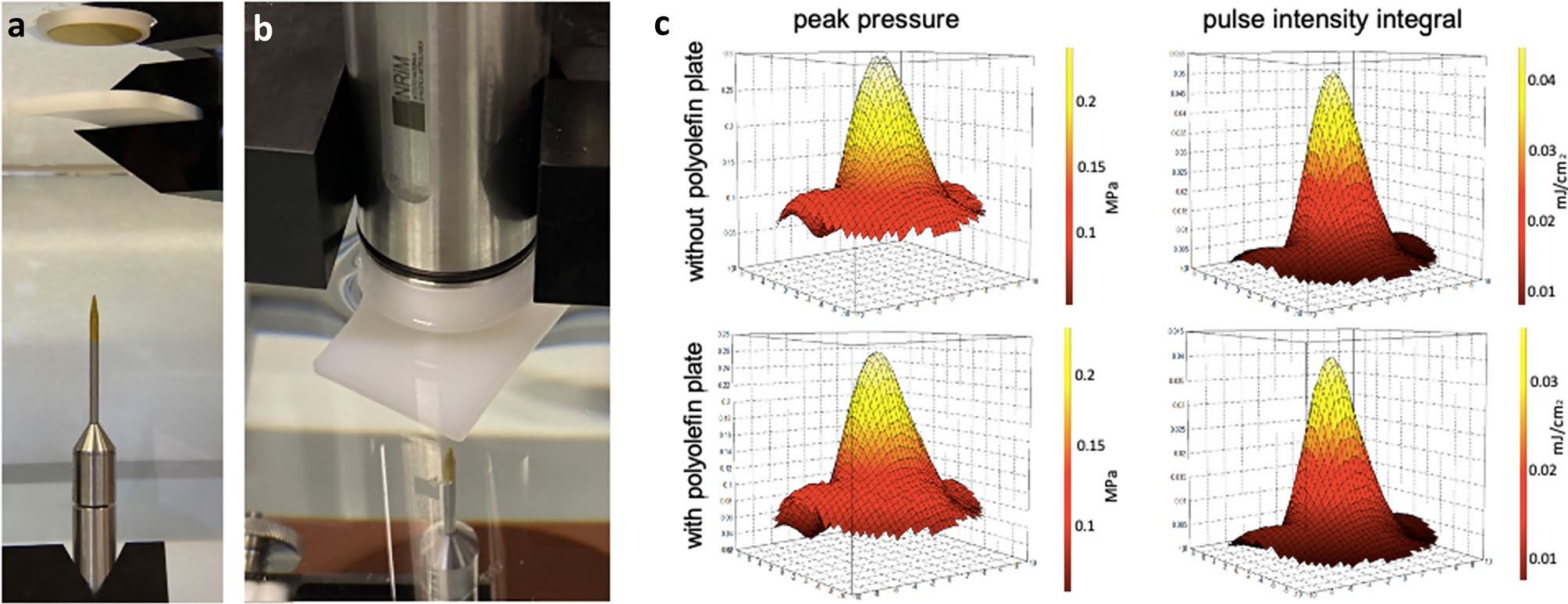
Acoustic characterization of the ultrasound-transparent polyolefin plate. (**a,b**) Measurements setup: (**a**) from top to bottom: surface of TRA08 transducer, polyolefin plate, HNC-0400 hydrophone. (**c**) Comparisons between two-dimensional measured array pressure (left panels) and energy (right panels), with polyolefin plate placed at 15 mm from the transducer surface (lower panels) and without polyolefin plate (upper panels).

Preliminarily to the BBB-permeabilization experiments, the passage of the US waves through the polyolefin material was characterized in INRiM Ultrasound laboratory (Turin, Italy); for this part of the study, the polyolefin plate, the ultrasonic instrumentation and the insonation protocol under examination were identical to those employed for the experiments on the in vitro brains (detailed below). Planar scans were performed around the transducer with and without an interposition of a 4 mm polyolefin plate placed at z = 15 mm from the transducer surface, see Fig. 1a,b.

After transducer mounting in fixed point, the alignment of the hydrophone in the direction of maximum sensitivity was carried out. Automatic search to locate the point of maximum spatial peak acoustic pressure was performed by scanning the volume in which the ultrasonic field is produced. The hydrophone was placed on the plane, which was 75 mm away from the transducer surface. The hydrophone moves in the x–y-axis (lateral directions) with step of 1 mm. For each point of the plane (20 mm × 20 mm) the oscilloscope acquired voltage waveforms, V(t), with a sampling frequency of 2 GSample/s for a time period of 10 μs, and one acoustic pressure was measured. Using the 400 measured points an ultrasonic emitted field is obtained (Fig. 1c).

The US pressure and energy were evaluated on hydrophone voltage waveform acquired by a digital oscilloscope and post-elaborated on the PC. Planar scans (20 mm × 20 mm), at 75 mm away from the transducer surface, were performed with and without an interposition of a 4 mm polyolefin plate placed at z = 15 mm from the transducer surface (Fig. 1). The results obtained at 0.3 MPa showed differences no higher than 12% in terms of pressure levels (left panels in Fig. 1c) and energy (right panels in Fig. 1c) between the scans performed with the interposition of the polyolefin plate and the ones acquired without. No significant planar shifts in x or y of the US beam could be observed between the same groups.

### US imaging of the isolated guinea pig brain

**Figure 2:**
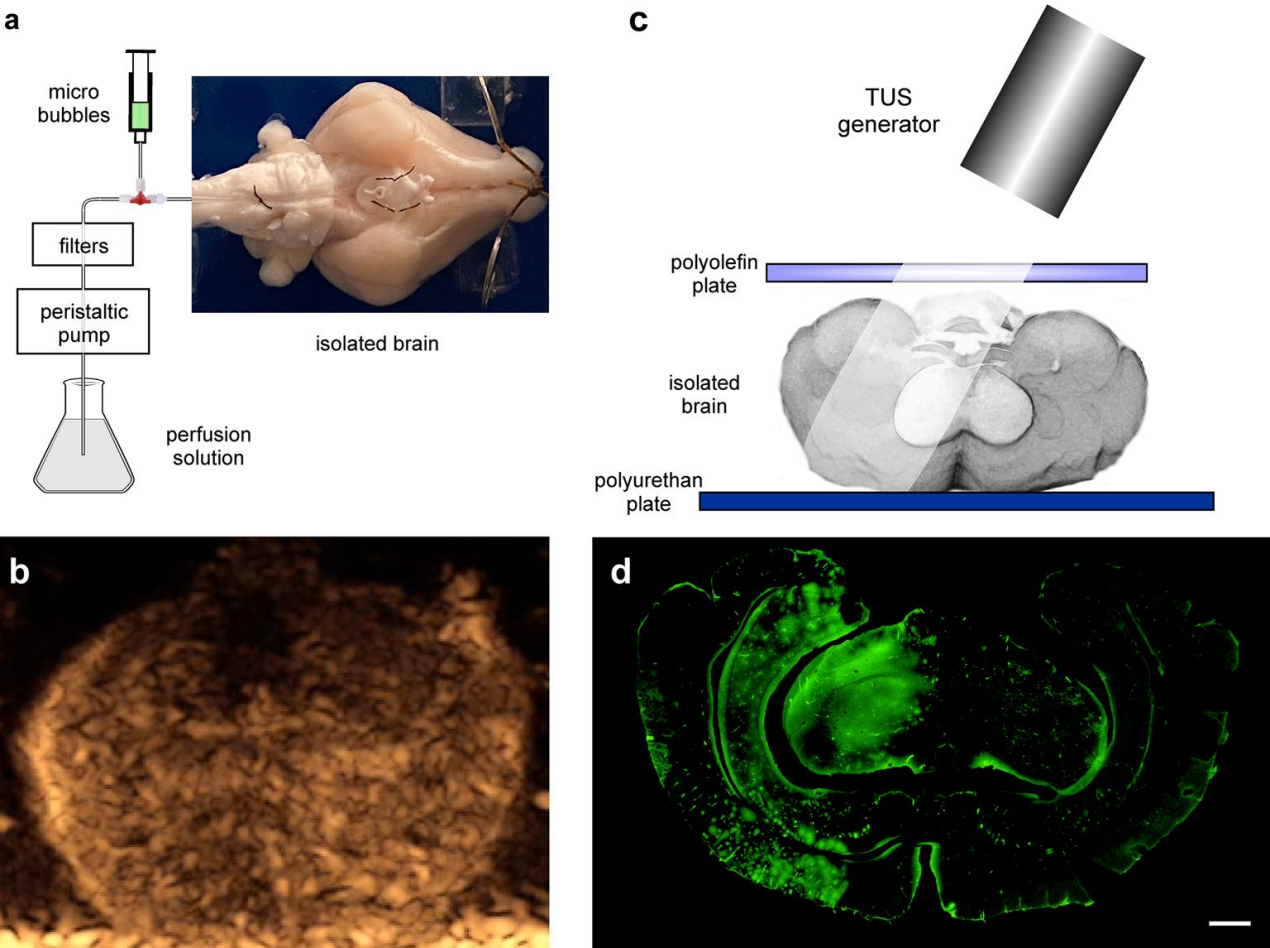
Scheme of the experimental setup for trans polyolefin plate MBs + TUS-mediated BBB opening. (**a**) Scheme of the experimental setup for microbubble (MBs) perfusion via the resident arterial system of the in vitro isolated brain preparation (photo on the right). MBs are injected via a syringe tributary of the main perfusion line. (**b**) Photograph of a coronal section obtained from CEUS imaging for the visualization of MBs circulation in the isolated guinea pig brain (see also Supplementary Movie 1). (**c**) Scheme of the sonication set up. MBs sonication was performed using a US generator; the area of influence of TUS is limited to one hemisphere. The polyolefin plate was interposed between the transducer and the brain surface. A US-absorbing polyurethane plate was positioned at the bottom of the incubation chamber to prevent US waves reflection. (**d**) Representative photomicrograph of a coronal brain section showing unilateral intra-cerebral FITC–albumin signal following TUS stimulation protocol during MBs infusion. Wide areas of FITC–albumin parenchymal extravasation indicating enhanced BBB permeability were detected in the left hemisphere regions affected by the TUS stimulation. The upside-down position of the brain and the spatial distribution is representative of the experimental setting in the recording chamber. Calibration bar = 100 µm.

Real-time visualization of the circulation and distribution of MBs perfused in the resident cerebrovascular system of the in vitro isolated brain preparation could be verified in a preliminary subset of experiments using a US diagnostic device equipped with Contrast Tuned Imaging (CnTI) algorithm (Fig. 2b, Supplementary Movie 1). Qualitative imaging analysis, assessed by a neurosurgeon with specific training on CEUS imaging (FP), showed effective MBs circulation within the brains both with and without the interposition of the 4 mm polyolefin plate (n = 3 and n = 3, respectively). A steady MBs concentration was generally achieved about 10 s after first MBs visualization. Since the isolated brains lack venous drainage, continuous infusion allowed a homogenous distribution of MBs across the entire duration of the scan. No significant differences were reported in scans performed with the prosthesis compared to the ones without its interposition. The polyolefin plate was visualized as an anechoic layer with hyperechogenous margins overlying the brain, replicating the findings from a previous in vivo experience with the same device^23^ (Fig. 2b).

### US-mediated BBB disruption

**Figure 3:**
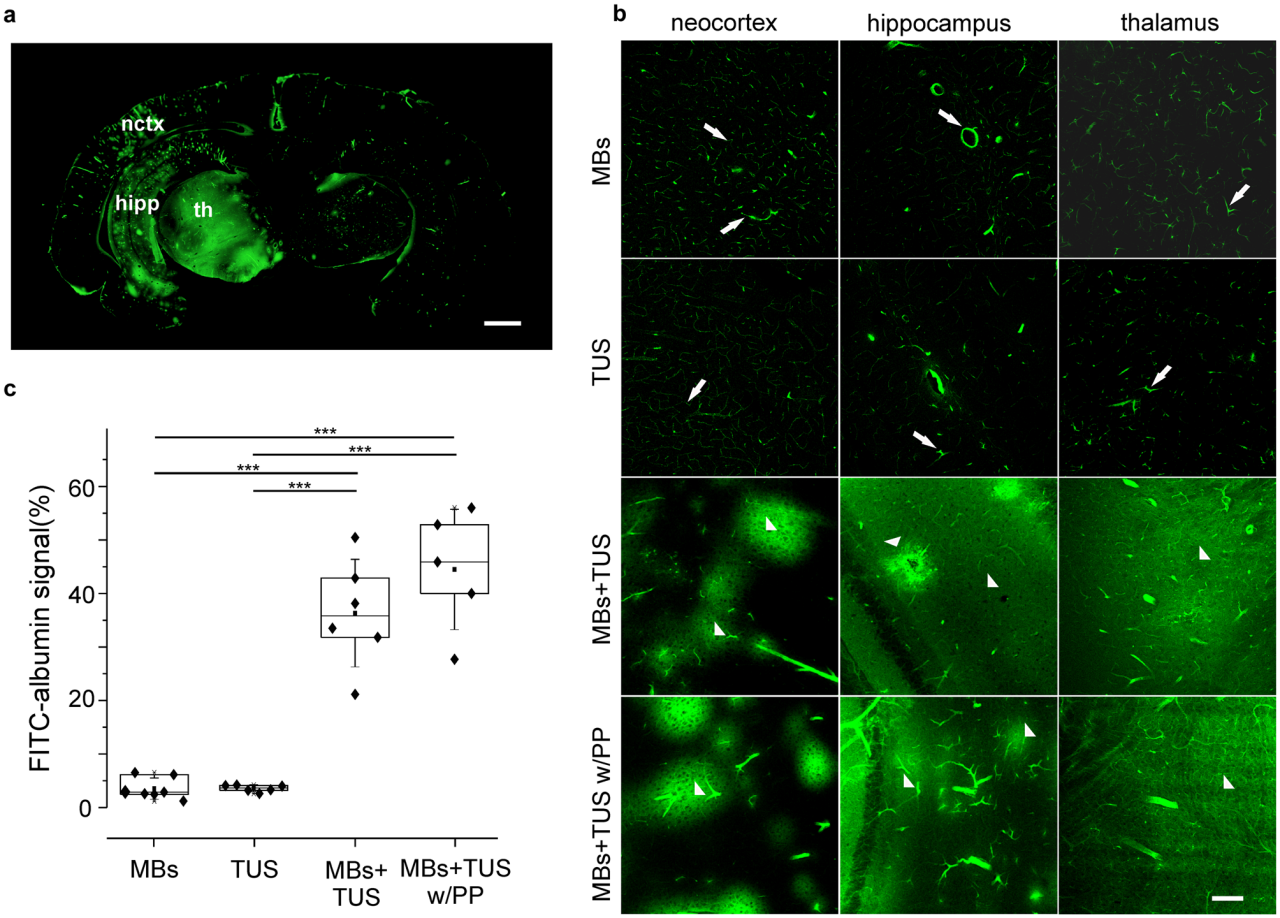
Polyolefin plate interposition consents MBs + TUS induced BBB-opening. (**a**) Representative photomicrographs of a coronal brain section showing the intra-parenchymal FITC–albumin signal following TUS stimulation performed on the left hemisphere during MBs infusion. The somatosensory neocortex (nctx), the hippocampus (hipp) and the thalamus (th) of the left hemisphere were *treated with* TUS stimulation. (**b**) High resolution confocal microscope photomicrographs of FITC–albumin signal in neocortex, hippocampus and thalamus in control conditions (MBs or TUS only first and second rows, respectively) and after the application of the MBs + TUS, with (bottom row) or without (third row from the top) the interposed polyolefin plate (PP). Under control conditions, no BBB damage occurred. Brain sections showed exclusively intraluminal signal with scattered perivascular spots (white arrows). Areas of a wide FITC–albumin parenchymal extravasation around vessels (white arrowheads) were detected after the application of MBs + TUS with or without the *polyolefin plate*. Calibration bar = 100 µm. (**c**) Quantification of FITC–albumin leakage in the four experimental conditions reported in the “Methods” section. FITC-albumin signal was quantified measuring fluorescence density, expressed as area occupied by the FITC-albumin. ANOVA followed by post hoc Tukey’s test was performed to compare the measurements obtained in the four experimental conditions, ***p ≤ 0.001.

The infusion of MBs or the exclusive application of TUS without MBs did not alter the BBB permeability in the in vitro brain preparation. The fluorescent signal generated by FITC-albumin was confined into the vessels (n = 13; intraluminal, right hemispheres in Figs. 2d, 3a and two upper rows in Fig. 3b,c) in the hemisphere not exposed to TUS, whereas unilateral leakage of arterially-injected FITC–albumin into the brain parenchyma was observed in the hemispheres that received TUS stimulation during MBs infusion (n = 11; left hemispheres in Figs. 2d, 3a; third row in Fig. 3b,c). FITC-albumin extravasation was similar in areas that received only MBs or TUS stimulation protocol (p = 1 and q = 0.007, respectively, from post hoc Tukey test). These findings demonstrate that TUS-mediated enhancement of BBB permeability can be achieved only by coupling MBs infusion and TUS.

Measurements of BBB changes analysed by one way ANOVA, followed by post hoc Tukey test, confirmed the BBB permeability increase when MBs and TUS were co-applied with or without the polyolefin plate, compared to control conditions characterized by both separate infusion of MBs and exclusive TUS stimulation: F(3) = 51.54; p = 1.36E−9 and p = 4.6E−7 and q = 11.6 for MBs vs TUS + MBs and p = 8.68E−7 and q = 11.1 for TUS vs TUS + MBs; p = 4.7E−6 and q = 13.6 for MBs vs polyolefin plate and p = 4.8E−7 and q = 13.1 for TUS vs TUS + polyolefin plate). The areas of FITC-albumin extravasation measured were 0.6 ± 0.16 mm^2^ and 0.7 ± 0.2 mm^2^, respectively in brains in which TUS was delivered either without (Fig. 3c; n = 6) or with the interposition of the polyolefin based plate (Fig. 3c; n = 5). No significant difference in the area of FITC-albumin extravasation was observed between cerebral areas that received BBB opening protocol with or without the interposition of the polyolefin-based plate (Fig. 3; p = 0.3 and q = 2.54). These findings prove that TUS is effective through a 4 mm plate of artificial acoustic-permeable material (Fig. 3a,c; bottom row in Fig. 3b; see also Fig. 2c).

## Discussion

The present study demonstrates the feasibility of disrupting the BBB across a sonolucent device specifically designed for the unhindered penetration of US waves. Brain areas exposed to concomitant sonication and MBs administration displayed a fluorescent pattern compatible with FITC extravasation, with no significant differences dependent on the interposition of the polyolefin plate; on the other hand, brains separately exposed to either MBs or US waves only did not show BBB disruption. Moreover, no signs of acute tissue damage attributable to the treatment could be found in the brain specimens: We also demonstrate that US waves do not induce per se any sign of BBB disruption demonstrated by the restriction of FITC signal to the vessels. Interestingly, brain anatomy and MBs circulation were easily visualized through the ultrasonography equipment; furthermore, it was possible to observe real-time changes in CEUS pattern during insonation (see Supplementary Movie 1).

The use of the in vitro isolated guinea pig brain preparation facilitates the preclinical testing of US-induced BBB changes. The isolated brain provides a whole functioning brain with intact BBB to be insonated and imaged without the impediment and possibly confounding presence of the cranial bone. The direct infusion through the arterial line and the absence of a physiological circulation with blood cells consents a controlled perfusion with MBs and FITC-albumin that is not achievable in vivo. On the other hand, the experimental condition limits the possibility of evaluating potential tissue damages caused by the insonation procedure.

The use of the in vitro preparation has also some limitations. The in vitro nature of the study could be considered an intrinsic limitation, as it removes many possibly confounding factors that make up a more complex in vivo model; indeed, the in vitro whole brain model lacks venous drainage, not allowing recirculation of MBs, and is perused by a saline solution instead of full blood. Nevertheless, the definitive demonstration of the polyolefine material to consent US-MB mediated changes in BBB permeability could be achieved with the necessary controls exclusively in the facilitated condition provided by the in vitro setting. In vivo experiments to test US-induced MB would present critical complexities and limitations. The controlled brain deliver of MB and FITC-albumin is easily achieved in vitro, whereas it may be more difficult and unreliable when both compounds are delivered by intravenous perfusion in vivo. In addition, the surgical implant of a polyolefin prosthetic plate in vivo (by itself a complex task) could induce a local inflammatory response that may alter BBB permeability in the region of the implant. BBB permeability changes could also be induced by the extraction of the brain after an in vivo experiment, a procedure that by itself would induce FITC-albumin extravasation that could be a confounder for the detection of specific US-MB-mediated effects. The size of the guinea pig brain does not allow for precise imaging of single structures with an ordinary clinical ultrasonography device; these limitations may be easily overcome by future studies employing larger subjects in vivo, also providing the means for an actual correlation of effects to CEUS enhancement patterns, assessed in a quantitative manner, correlated to the BBB outcome.

The presented findings represent the necessary preliminary step to propose polyolefin-based material as US-permeable skull prostheses to be used for repetitive BBB openings in a clinical setting. The concept of implanting sonolucent cranioplasties for imaging purposes has already been fostered by various authors^27–29^; most recently, two articles were published by Jan-Karl Burkhardt’s group proposing such concept to be exploited in the follow-up of intracranial bypasses with satisfactory safety and efficacy^30,31^. However, only one article from 1987 had theorized the implantation of US-permeable cranioplasty materials for TUS purposes: Tobias and collaborators already proposed US-based ablative treatments to be performed across a sonolucent window to preserve the mechanical protection of the skull while allowing full penetration of the US waves^32^; in their study, these authors proved that homogenous and low-porosity materials cause the least attenuation and distortion of the incident beam. However, their perspective only envisaged the evaluation of these materials for therapeutic purposes involving FUS without the possibility of guiding such procedures with ultrasonography^32^.

In the neurosurgical practice, US waves are ordinarily employed for imaging purposes, exploiting the real-time, multimodal feedback during surgical procedures; while standard B-Mode provides the surgeon with morphological details on both physiological and pathological anatomy, including information on tumor residue, other modalities yield functional information on the inspected tissue, including texture by calculating Young’s modulus with sonoelastographic imaging and vessels’ flow with Doppler techniques^33,34^. CEUS imaging on the other hand exploits the purely intravascular nature of MBs to provide detailed overview of tissue’s perfusion regardless of the probe’s orientation^8^. Indeed, evaluation of CEUS imaging for intraoperative imaging is mainly qualitative, but different studies have highlighted differences in enhancing patterns when different structures are observed: for example, Prada and collaborators described a difference in timing and intensity of signal between different brain areas and brain tumors, with the possibility of making differential diagnosis between low- and high-grade tumors^35^. Such qualitative differences can be translated into an in-depth, quantitative analysis, mainly by employing software to obtain time-intensity curves relatable to the various enhancing phases of structures, from arterial to capillary phases to venous washout^36^. An article was recently published describing quantitative differences between tumor lesions and surrounding structures, encompassing cortical and white matter, arteries and basal ganglia^37^. Regarding treatment, such information may be pivotal for the planning of therapeutic procedures involving MBs perfusion, in order to maximize effects to targets and sparing physiological structures without pinpointing the US waves to a single spot. Previous studies have already found the correlation of MBs features with the degree and timing of BBB disruption; as an example, MBs’ size has been reported by numerous authors to be directly correlated to the outcome of BBB, with larger MBs producing significant effect at lower acoustic pressures compared to smaller MBs^18,38,39^; also the administered dose and molecular composition of MBs have been found to affect BBB opening^40–42^. Quite recently, Song et al. postulated that total gas volume may be used as a unifying parameter to plan focused-ultrasound (FUS) treatments^43^.

Interestingly, one of these authors also advocated for continuous infusion of MBs to be employed for treatments in order to achieve a stable concentration of MBs within insonated tissues^42^.

Overall, US-mediated BBB disruption might benefit from both a wide-radius, unfocused approach, exploiting the “auto-focusing” properties of MBs when exposed to US waves, and a contrast-enhanced, US-based planning and real-time feedback, allowing to adapt insonation parameters to the pattern of MBs enhancement of target tissues. The issue of tracing MBs is not new in the field of US-mediated BBB disruption: throughout the years, different authors advocated the use of passive cavitation detectors to trace the harmonic emissions of insonated MBs during insonation procedures; the efforts of these authors resulted in passive acoustic mapping techniques, in which receiver-only arrays of single or multiple elements are able to spatially translate cavitative phenomena and to distinguish them between stable and inertial cavitation, indicative respectively of reversible, non-disruptive BBB opening or noncontrollable tissue injury due to MBs’ collapse^19,44,45^. However, such systems may only provide information during sonication, thus not allowing planning of the treatment based on MBs visualization and quantification at baseline immediately before treatment. While current FUS therapies are applied through MR-guided devices, their use for BBB opening, which encompasses larger volumes compared to micro-ablative techniques, has already been questioned^46^; indeed, the large size, costs and treatment times of these devices may prevent the large application of BBB-disruptive treatments, especially for older patients and if repeated sessions are required to achieve significant clinical results. In recent years, various authors attempted to solve this issue either by administering FUS through navigated, stereotaxic systems, which maintain the accuracy of the much larger MR suites, or by implanting unfocused US devices into the patients’ cranial convexity, which on the other hand bypass the need of cumbersome settings while also forgoing any kind of monitoring and adjustability of the treatment^12–15,47^. The application of US waves through a permeable polyolefin plate envisaged in this study might represent a good synthesis of these approaches, allowing for US guided therapeutic stimulation. Indeed, ultrasonography is traditionally applied bedside to the patient without the need of a dedicated environment, but the uncoupling of the polyolefin plate and the insonation means would allow close monitoring of the treatment; as for accuracy of the treatment, nowadays it is already possible to navigate US probes and to superimpose US imaging to both MR and CT pre-acquired scans in real time^34^.

An efficient sonolucent cranioplasty might change the perspective of US-mediated transient BBB disruption and, globally, of all TUS treatments requiring a wide sonication radius and repeated sonication sessions. Neuro-oncology provides a perfect example, as skull replacements are already largely employed^20^; in this field, pharmacological therapies require multiple and closely planned sessions, the tight schedule of which would hinder the systematic application of current US-mediated BBB-disruption systems. The potential advantages of repeated BBB opening along chemotherapy have already been largely debated, and preliminary clinical experiences have already shown significant survival benefits in patients achieving BBB disruption, verified at post-insonation MRIs^17^. However, the potential fields of application of this design are much broader than neuro-oncologic diseases: repeated BBB disruption might be used in the future to deliver growth factors or gene therapy to the CNS^48,49^, as well as to enhance clearance of toxic substances like beta-amyloid in Alzheimer disease^50,51^; all of which may benefit from a more tailorable and flexible approach, while leaving focused technology to applications which need the highest precision and accuracy of US waves delivery, such as ablative treatments or BBB disruption-enabled liquid biopsies^48^. In addition to neuro-oncology, further potential fields of application of US-guided therapeutic ultrasound encompass neurotrauma, neurodegenerative pathologies, movement disorders, brain ischemia and many others^48^.

In conclusion, the results of the present study clearly demonstrate that TUS-mediated BBB permeabilization is achievable through an artificial sonolucent window, providing preliminary preclinical evidence for future repeated US-guided ultrasound applications for BBB opening. A CEUS-guided method for BBB permeabilization through an implanted polyolefin plate prosthesis is desirable to achieve a bedside, repetitive, adjustable and accessible approach to enhance pharmacotherapy for many pathologies of the CNS. The preliminary step achieved through the present study strengthen the potential clinical relevance of the use of polyolefin-based prostheses in neurosurgery.

## Methods

### Study design

In the present experimental design, 24 hemispheres from 12 guinea pig brains were analysed. Treatment groups included: TUS only (n = 6); MBs only (n = 7), MBs mediated BBB opening by TUS with (n = 5) or without (n = 6; Fig. 2c) the interposition of a 30 × 60 × 4 mm, 0.93 g/cm^3^ density and 0.44 g/cm^3^ porosity rectangular plate of US-transparent polyolefin material between the transducer and the brain surface. In 5 out of 12 brains, BBB opening was performed in one hemisphere and the contralateral was considered either as MBs or TUS exclusive treatment.

### Acoustic characterization of the polyolefin plate

For the evaluation of polyolefin plate it has been used a plane wave ultrasound transducer, TRA08, INRIM made based on lithium-niobate piezoelectric transducer (BOSTON PIEZO-OPTICS INC), with a center frequency of 0.985 MHz), connected with signal generator (Agilent model 33250A) via power amplifier (AMPLIFIER RESEARCH model AR 100A250A). Generator trigger pulse output, synchronized with the signal excitation of the transducer, was connected to the oscilloscope of the scanning tank system.

The ultrasound measurements took place in a plexiglass tank filled with distilled water using scanning tank system (ONDA corporation model AIMS III). Tank dimensions were: length 1.0 m, width 0.5 m, height 0.6 m. Measurements were conducted by a needle hydrophone (ONDA Corporation Model HNC-0400) connected with pre-amplifier (ONDA Corporation Model AH-2020); the hydrophone was mounted on an adjustable support linked with three linear actuators. The actuators were driven by a stepper motor giving a positional resolution of 20 μm.

The definition of the ultrasound field parameters, pressure and energy, is based on hydrophone voltage waveform, V(t), acquired by a digital oscilloscope and post-elaborated on the PC.

A deconvolution process was applied to obtain the pressure waveform, p(t), using the sensitivity curve of the hydrophone, M(f):

$$ p(t) = \Im^{ - 1} \left\{ {\frac{{\Im \left\{ {V(t)} \right\}}}{M(f)}} \right\}, $$

where $\Im \left\{ {V(t)} \right\}$ and $\Im^{ - 1} \left\{ {V(\omega )} \right\}$ → Fourier transform and inverse-transform; $M(f)$ → Frequency response of the hydrophone obtained from its calibration certificate.

### In vitro brain preparation

Brains were isolated from young adult female Hartley guinea pigs (150–200 g; Charles River Laboratories) according to the standard technique described in details elsewhere^24^. After barbiturate anaesthesia (sodium thiopental, 125 mg/kg, i.p.), guinea pigs were transcardially perfused using a peristaltic pump (Gilson Minipulse 4) with a cold (15 °C) oxygenated (95% O_2_/5% CO_2_; pH 7.1) saline solution composed by NaCl, 126 mM, KCl, 3 mM, KH_2_PO_4_, 1.2 mM, MgSO_4_, 1.3 mM, CaCl_2_, 2.4 94 mM, NaHCO_3_, 26 mM, glucose, 15 mM, 3% hydroxyethyl starch (Voluven, Fresenius Kabi) with M.W. of 130 KDa. Cardiac perfusion removes blood from cerebral vessels and reduce brain metabolism to preserve the brain tissue from hypoxia during the dissection. The animal is decapitated after 3 min, and the brain is carefully isolated and transferred to the incubation chamber. The bottom of the chamber is coated with a US-absorbing, micro-bubble filled, pre-cast polyurethane plate (AptFlexF28, Acoustic Polymers Ltd; Fig. 2c). After dissection, the isolated brain is positioned in the incubation chamber with its ventral surface upward to visualize the base of the brain and the resident vascular system. The olfactory bulbs and the cervical spinal cord are held down by two silk threads for mechanical stabilization. Under a stereomicroscope, the dura mater that enfolds the basilar artery is gently removed, and a polyethylene cannula (terminal gauge 0.25 mm) is inserted in the basilar artery. The cannula is tied to the basilar artery with a silk thread and the brain circulation is restored by arterial perfusion with the above solution (pH 7.3) at a rate of 6 ml/min via a peristaltic pump (Gilson Minipulse 4) connected to a set of tygon tubing. Then, the carotid and the hypophyseal arteries are ligated with silk knots to re-establish the physiological cerebral vascular flow through the Willis circle without arterial leaks. The temperature of the incubation chamber is successively increased from 15 to 22 °C (room temperature), at a rate of 0.2 °C min^−1^, with a controlled ramp to heat exchangers (Peltier devices) commanded by a thermostat connected to a temperature controller (NPI, RG-01). Carefully isolated in vitro guinea pig brains show close to- physiological activity for 6–8 h.

The brain isolation procedure was conducted in accordance with the guidelines defined by the European Communities Council directive (2010/63/EU), ARRIVE guidelines and RRR principles. Efforts were made to limit the number of animals used. The experimental protocol was reviewed and approved by Committee on Animal Care and Use and by the Ethics Committee of the Fondazione IRCCS Istituto Neurologico *Carlo Besta* (DO-01-2020) and by the Italian Ministry of Health (Aut. Min. No. 271-2012/A).

### MBs circulation

MBs (SonoVue, Bracco, Italy) were used to provide the cavitating gas for US-mediated BBB disruption^52^. MBs provided as a sulphur hexafluoride powder were reconstituted in 10 ml of 0.9% NaCl solution (0.5–2.5 × 108 MBs/ml) and were perfused into the isolated guinea pig brain at the rate of 1 ml/min via a syringe-infuser (KD Scientific) connected to the main arterial perfusion line (Fig. 2a). Continuous infusion was preferred over bolus administration to provide homogeneous MBs distribution.

MBs circulation within the in vitro brain was assessed in a preliminary subset of experiments using a portable US system (MyLab Omega, Esaote SpA, Italy) equipped with a dedicate algorithm (Contrast Tuned Imaging, CnTI) for CEUS. A 3–11 MHz linear-array diagnostic probe was placed on a holder perpendicular to the brain axial plane; the whole brain was scanned in coronal sections (Fig. 2b, Supplementary Movie 1). Imaging was performed both with and without the polyolefin plate in place. CEUS imaging was displayed via the inbuilt screen recorder of the device; imaging was started at the beginning of MBs arterial infusion.

### Sonication protocol

Sonication of MBs for BBB opening was performed using a planar transducer with nominal frequency of 0.989 MHz placed over the targeted hemisphere, sonicating a region that included one temporal lobe (Figs. 2d, 3a), for 2 consecutive minutes with intensity from 20 to 100 mW/cm^2^, pulsed for 100 ms ON, 900 ms OFF duty cycle (1 kHz pulse repetition rate). In 5 experiments a polyolefin-based plate was interposed between the transducer and the isolated brain. The US transducer was connected to an amplifier (E&I model 350L) and to a function generator (Agilent model 33500B). The BBB-opening sonication protocol started 1 min after MBs infusion. At the end of sonication protocol, the MBs infusion was interrupted and a 5-min washout with standard solution followed.

### Evaluation and quantification of BBB permeability changes

Previous studies demonstrated the functional integrity of the BBB in the isolated guinea pig brain for several hours during in vitro incubation, as shown by the analysis of the molecular organization of the microvessels and the endothelial junctional complexes that are primary components of the BBB^53^. The presence of BBB breakdown in isolated brains was assessed by perfusing fluorescein isothiocyanate (FITC)–albumin (50 mg/10 ml, Sigma- Aldrich) for 4 min at the end of the sonication experiment. Brains were rapidly removed from the incubation chamber, the cerebellum and the olfactory bulbs were excised, and were fixed by immersion for 48 h in 4% paraformaldehyde in phosphate-buffered saline (PBS; 0.1 M, pH 7.4)^53^.

Coronal section (50 μm) were cut by vibratome (VT 1000S Leica) throughout the extension of the hippocampus (plates A5.4–A6.24 of the guinea pig brain atlas by Luparello^54^). Sections collected on gelatin-coated slides were mounted in Fluorosave (Calbiochem) and cover-slipped. Two representative sections corresponding to coronal rostrocaudal level A5.40 and A6.00 of the Luparello atlas were collected for each brain and used for the subsequent quantification. Slide-mounted sections were examined with a laser scanning confocal microscope D-Eclipse C1 (Nikon), equipped with three laser lines, mounted on a light microscope Eclipse TE2000-E (Nikon) using excitation light of 488 nm (Laser Ar). Laser intensity was set at 30–35% power. Gain and photomultiplier were kept constant during the acquisition of all images. Quantification of FITC–albumin fluorescent signal was performed in the neocortex, in the hippocampal formation and in the thalamus. In each brain, five high-power non-overlapping fields (region of interest, [ROI] of 1.6 mm^2^ each) per section were acquired bilaterally at × 10 magnification, image size 512 × 512 pixels. The percentage of FITC–albumin signal (number of pixels) was estimated and quantified using the Image-Pro Premier 9.1 software (Media Cybernetics). An average of coefficients obtained from the examined fields was calculated and the statistical analysis was performed. Sections were also scanned with the Nanozoomer HT (Hamamatsu Photonics K.K) at × 10 magnification with a resolution of 443 μm/pixel in three z-layers with a spacing of 2 μm each. For each region the best focused z-layer was determined and saved as an image to be available for the following segmentation and profiling algorithms. The data measured in the 5 non overlapping fields per slice were averaged, providing a single value for each hemisphere that was used for statistical analysis of data.

### Statistical analysis

Quantitative results were analysed using one-way analysis of variance ANOVA followed by Tukey post-hoc test. The normal distribution of samples was checked with Shapiro–Wilks test. All statistical tests were performed with Origin Pro2016 (OriginLab Corporation). The format for ANOVA test was F(df) = F, p = significance value and p value. The tests are two-sided and confidence interval of 95% (p < 0.05) was required for values to be statistically significant. Data are shown as mean ± standard deviation (SD).

### Ethics committee approval

The experimental protocol was reviewed and approved by Committee on Animal Care and Use and by the Ethics Committee of the Fondazione IRCCS Istituto Neurologico Carlo Besta (DO-01-2020) and by the Italian Ministry of Health (Aut. Min. No. 271-2012/A).

## Supplementary Information

Supplementary Legends.

Supplementary Video 1.
