# Supplementary material for: Ultrasounds induce blood–brain barrier opening across a sonolucent polyolefin plate in an in vitro isolated brain preparation
Source: Sci Rep. 2022 Feb 21;12:2906. doi: 10.1038/s41598-022-06791-7 (PMC8861168; doi:10.1038/s41598-022-06791-7)
Supplement: Supplementary file 1 — Supplementary Legends. [file 41598_2022_6791_MOESM1_ESM.docx]

**CEUS imaging of MBs circulation in the *in vitro* isolated guinea pig.**

Brain positioned with the dorsal part on the US-absorbing polyurethane plate at the bottom of the recording chamber. The video recording was performed with a linear probe imaging the brain in a coronal plane at the level of the optic chiasm (see **also Figure 2a and b**). Arterially perfused MBs enter the brain at 6 seconds.
